# Investigation of a multicomponent mycotoxin detoxifying agent for aflatoxin B1 and ochratoxin A-induced blood profile in broiler chickens

**DOI:** 10.14202/vetworld.2024.1044-1051

**Published:** 2024-05-15

**Authors:** Mutmainah Wardatul Jannah, Fitri Handayani, Bambang Sektiari Lukiswanto, Mohammad Anam Al Arif, Suwarno Suwarno, Hery Purnobasuki, Rahmi Sugihartuti, Suzanita Utama, Siti Darodjah, Tita Damayanti Lestari, Mirni Lamid, Goo Jang, Erma Safitri

**Affiliations:** 1 Department of Veterinary Medicine, Student of Veterinary Medicine Faculty, Universitas Airlangga, Surabaya, East Java, Indonesia; 2 Veterinary Clinic Division of Veterinary Medicine Faculty, Universitas Airlangga, Surabaya, East Java, Indonesia; 3 Veterinary Animal Husbandry Division of Veterinary Medicine Faculty, Universitas Airlangga, Surabaya, East Java, Indonesia; 4 Veterinary Microbiology Division of Veterinary Medicine Faculty, Universitas Airlangga, Surabaya, East Java, Indonesia; 5 Department of Biology, Faculty of Science and Technology, Universitas Airlangga, Surabaya, East Java, Indonesia; 6 Basic Veterinary Medicine Division of Veterinary Medicine Faculty, Universitas Airlangga, Surabaya, East Java, Indonesia; 7 Veterinary Reproduction Division of Veterinary Medicine Faculty, Universitas Airlangga, Surabaya, East Java, Indonesia; 8 Department of Animal Production, Animal Husbandry Faculty, Universitas Padjadjaran, West Java Indonesia; 9 Department of Theriogenology, College of Veterinary Medicine, Seoul National University, Seoul, Republic of Korea

**Keywords:** blood profile, broiler, healthy, mycotoxin detoxifier, mycotoxin

## Abstract

**Background and Aim:**

Mycotoxins such as aflatoxin B1 and ochratoxin A (OTA) are secondary metabolites in molds that grow in raw materials or commercial feed. This interaction has a synergistic effect on mortality, body weight, feed intake, embryo abnormalities, egg production, and lymphoid organ atrophy. This study was conducted to determine the effect of a mycotoxin detoxifier on the blood profile of broilers that were given feed contaminated with mycotoxin, such as the number of heterophils, lymphocytes, monocytes, mean corpuscular hemoglobin (MCH), and MCH concentration (MCHC).

**Materials and Methods:**

A total of 20 day-old chicks (DOC) of Cobb broilers were given four treatments with five replicates. The number of chickens used in this research was determined using statistical calculations, and the data obtained was homogeneous so that the population was represented. Treatments included negative control with basal feed (C-), positive control with mycotoxins contamination (C+), treatment 1: Mycotoxins contamination and mycotoxin detoxification 1.1 g/kg (T1), and treatment 2: Mycotoxins contamination and mycotoxin detoxification 1.6 g/kg (T2). Mycotoxin contamination comprised 0.1 mg/kg aflatoxin B1 and 0.1 mg/kg OTA. The treatment period for chickens was 28 days, from 8 to 35 days. A battery cage was used in this study. Chickens were kept in a closed, ventilated room and the room temperature (27°C) was monitored during the treatment period.

**Results:**

Based on the results of statistical data processing, a significant difference (p < 0.05) was observed between chickens fed mycotoxin-contaminated feed (C+) and chickens not fed mycotoxin-contaminated feed (C-) and chickens given 1.6 g/kg mycotoxin detoxification (T2). Mycotoxin detoxification at a dose of 1.6 g/kg had a significant (p < 0.05) effect on the heterophil, lymphocyte, and heterophil lymphocyte ratio, leukocyte, erythrocyte, and hemoglobin levels of the blood broiler in this experiment. On other parameters such as monocytes, MCH, and MCHC, treatment 2 at dose 1.6 g/kg was the best treatment, although there was no significant effect with C- and T1.

**Conclusion:**

The administration of mycotoxin detoxifiers at a dose of 1.6 g/kg increased the number of heterophils and the ratio of heterophil lymphocytes, leukocytes, erythrocytes, and hemoglobin in broilers fed mycotoxin-contaminated feed.

## Introduction

Mycotoxins are secondary metabolites in certain molds that grow in raw material feed or commercial feed [1]. Mycotoxins have been reported to cause economic loss, disease in livestock and humans, and even death [2]. Five mycotoxins, aflatoxin, fumonisin, ochratoxin, trichothecene and zearalenone, have been reported to be dangerous to the body. Aflatoxins and ochratoxins are examples of mycotoxins whose toxicity levels are higher than others [3]. Aflatoxins and ochratoxins are often found together in grains where they cause severe damage due to their synergistic effects. The interaction between aflatoxins and ochratoxins has a synergistic effect on mortality, body weight, feed intake, embryo abnormalities, egg production, and lymphoid organ atrophy. One of the main concerns is a decrease in immunity in which the immune system of animals weakens [4].

To treat mycotoxicosis, mycotoxin detoxifier agents are required. A mycotoxin detoxifier is an ingredient that can be used as an alternative to treat mycotoxin contamination in feed. An alternative approach to reducing exposure to mycotoxins in animal feed is to reduce their bioavailability using a mycotoxin detoxifier [5]. A number of strategies have been developed to (1) reduce the growth of mycotoxigenic fungi and mycotoxin production, (2) detoxify contaminated feed, and (3) lower the systemic availability once mycotoxins are ingested by animals [5]. Mycotoxin detoxifiers can be classified into two sub-categories: Adsorbing agents or toxin binders and biotransforming agents or mycotoxin modifiers [6]. Binding of aflatoxin with the mycotoxin binder is based on the principle of polarity, whereby the negative polarity of the aflatoxin is bound by the positive polarity of the toxin binder so that the toxin can be mobilized and eliminated from the animal’s body [7]. In contrast to aflatoxin, which has polar molecular properties that can be treated with toxin absorbents, ochratoxin is a nonpolar molecule [8]. An alternative strategy for dealing with ochratoxins can be the use of mycotoxin modifiers, which can change mycotoxin structural molecules into less toxic or non-toxic metabolites [9].

As mentioned above, mycotoxin detoxifiers can be divided into two categories: Mycotoxin binders and mycotoxin modifiers. To date, research on the use of mycotoxin detoxifiers for the combination of mycotoxin contamination, such as aflatoxin B1 and ochratoxin A (OTA) of the blood profile, such as heterophile, lymphocyte, monocyte, mean corpuscular hemoglobin (MCH), and MCH concentration (MCHC) in poultry, is still rare.

This study aimed to determine the effect of a mycotoxin detoxifier on the blood profile of broilers fed feed contaminated with mycotoxin, such as the number of heterophils, lymphocytes, monocytes, mean corpuscular hemoglobin (MHC), and MCH concentration (MCHC).

## Materials and Methods

### Ethical approval

Chickens were handled humanely throughout the study, and the experimental design and protocol for the use of the chicken for research were approved by the Animal Care Use Committee of the Faculty of Veterinary Medicine Airlangga University (Approval No. 1.KEH.033.02.2023).

### Study period and location

This study was conducted from February 2023 and March 2023 at the Faculty of Veterinary Medicine, Airlangga University, Surabaya, Indonesia.

### Experimental animals and diets

In this study, 20 Cobb broiler day-old chicks were used as experimental animals. Chickens were reared from day old chick until day 35 and obtained from Charoen Pokphand Indonesia Company Limited. It was kept in deep litter with drinkers and standard feeding troughs. A basal diet was started from day-old chickens until 7 days old, and then, the experimental design was continued.

### Experimental design

A total of 20 chickens were divided into four treatment groups with five replications for each treatment. The chickens underwent an adaptation period until they were 7 days old. On the 8^th^ day, they were treated in stages according to a predetermined group. Treatments included negative control with basal feed (C–), positive control with mycotoxin contamination (C+), treatment 1: Mycotoxin contamination and mycotoxin detoxifier 1.1 g/kg (T1), and treatment 2: mycotoxin contamination and mycotoxin detoxifier 1.6 g/kg (T2). Mycotoxin contamination consisted of 0.1 mg/kg aflatoxin B1 and 0.1 mg/kg OTA. The feed used for rearing includes CP511 chicken feed produced by Charoen Pokphand Indonesia Company Limited and drinking water was provided *ad libitum*. Mycotoxins in the form of aflatoxin B1 (0.1 mg/kg), OTA (0.1 mg/kg), and the mycotoxin detoxifier Mycofix^®^ (Biomin GmbH, A-3130 Herzogenburg, Austria) were used for the treatment. Chickens were fed every morning and evening, with unlimited drinking water. Based on references to previous studies [10, 11], we have considered the use of mycotoxin detoxifier doses.

The choice of dose was chosen on the basis of the following considerations: According to Ramandani *et al*. [11], the level of aflatoxin that is still safe in feed ingredients is no more than 50 pbb or 0.05 mg/kg. Meanwhile, ochratoxin [10] at a dose of 0.1 mg/kg can already cause a decrease in the number of heterophiles. Based on these considerations, aflatoxin and ochratoxin doses of 0.1 mg/kg and 0.1 mg/kg, respectively, were chosen as the dose studied.

The cage used is a closed house equipped with a blower and a room thermometer to measure theroom temperature (27°C). Ventilation and lighting in the room were arranged in such a way as to support the health of broiler chickens. To avoid air pollution and vehicle noise, the cage used were placed far from major roads. The feed was stored in a dry and non-humid place to avoid damage to the feed ingredients. Cleaning of the cage was carried out every day by removing chicken droppings and changing the bedding to keep the cage clean.

### Blood profile with hematological determinations

Broiler chickens were to take blood samples on day 35. Chicken blood was collected through the brachial vein using a 1 mL syringe and accommodated in a 1 mL heparin tube [12]. The tube was inverted until it was homogeneous, then stored in a cool box and examined.

The blood profile was measured by collecting blood from the wing area in a heparin vacuum tube and then examining it using a hematology analyzer.

### Statistical analysis

Data obtained from the hematology analyzer test were then analyzed using the SPSS Version 20 (IBM Corp., NY, USA) and expressed in mean values and standard deviations [13] using the one-way ANOVA test [14]. If there is a difference between the treatments, it is continued with Ducan’s test with a significance level of 0.05 [15].

## Results and Discussion

**Figure-1:**
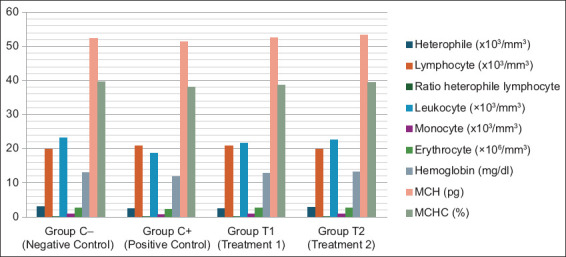
Blood profiles of aflatoxin B1 and ochratoxin A-induced broilers treated with mycotoxin detoxifying agent.

**Figure-2:**
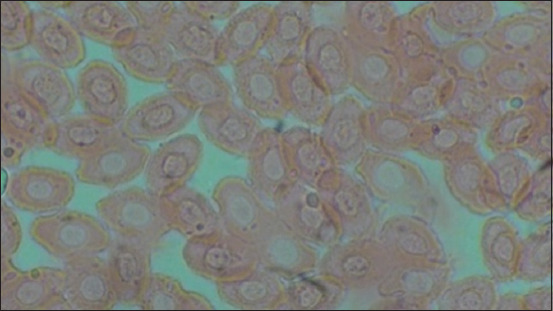
Negative control (C-).

**Figure-3:**
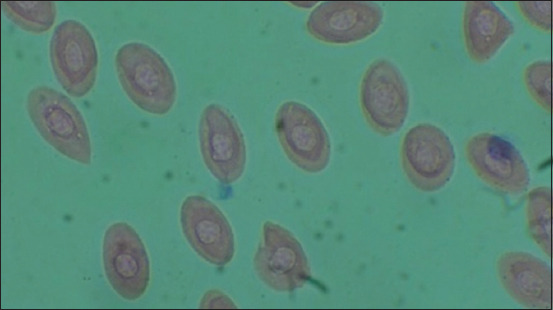
Positive control (C+).

**Figure-4:**
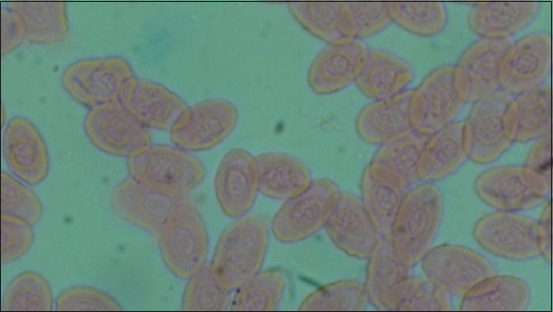
Treatment 1 (T1).

**Figure-5:**
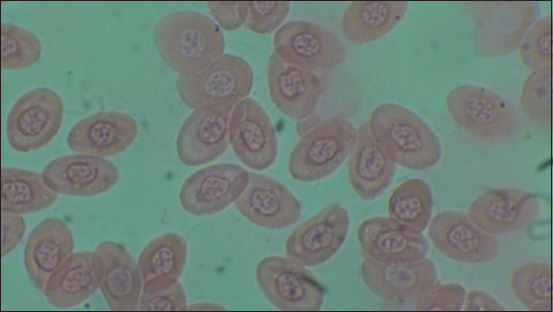
Treatment 2 (T2).

Table-1 and Figure-1 show the results of heterophile, lymphocyte, ratio heterophile lymphocyte, leukocytes, erytrocytes, hemoglobin, monocytes, MCH, and MCHC. The blood smear results are shown in Figure-2–5.

**Table 1:** Mean and standard deviation of broiler blood parameters.

Parameters unit	Group C− (negative control)	Group C+ (positive control)	Group T1 (treatment 1)	Group T2 (treatment 2)
Heterophile (×10^3^/mm^3^)	2.96ᵇ ± 0.20	2.40^a^ ± 0.39	2.54^ab^ ± 0.26	2.92ᵇ ± 0.37
Lymphocyte (×10^3^/mm^3^)	19.84^a^ ± 0.35	20.88^b^ ± 0.67	20.86^b^ ± 0.66	19.88^a^ ± 0.68
Ratio of heterophilic lymphocytes	0.149^b^ ± 0.012	0.115^a^ ± 0.020	0.121^a^ ± 0.014	0.146^b^ ± 0.018
Leukocyte (×10^3^/mm^3^)	23.180^b^ ± 1.10	18.620^a^ ± 1.30	21.660^b^ ± 1.93	22.630^b^ ± 0.95
Monocyte (×10^3^/mm^3^)	0.960^b^ ± 0.114	0.760^a^ ± 0.089	0.806^a^ ± 0.080	0.876^ab^ ± 0.122
Erythrocyte (×10^6^/mm^3^)	2.598^b^ ± 0.07	2.350^a^ ± 0.12	2.566^b^ ± 0.05	2.602^b^ ± 0.07
Hemoglobin (mg/dL)	13.070^b^ ± 0.57	11.830^a^ ± 0.37	12.780^b^ ± 0.46	13.190^b^ ± 0.58
MCH (pg)	52.4^a^ ± 1.23	51.3^a^ ± 1.89	52.5^a^ ± 1.84	53.3^a^ ± 1.85
MCHC (%)	39.6ᵇ ± 0.46	38.1^a^ ± 0.98	38.6^ab^ ± 0.42	39.5^ab^ ± 1.63

Different superscripts in the same line show differences significant (p < 0.05). MCH=Mean corpuscular hemoglobin, MCHC=Mean corpuscular hemoglobin concentration

Broiler chickens or meat chickens with high genetic quality are superior meat producers [16]. The survival of an organism depends on adaptation, natural selection, and reproduction [17, 18]. Chickens exposed to mycotoxins tend to suffer health problems. The following results were obtained based on the results of the research carried out. In the statistical analysis of the number of heterophiles, the positive control group (C+) was significantly different from the negative control group (C–) for 28 days. Heterophils are granulocytes that serve as the initial defense against disease [19]. Heterophils are the first line of defense activated during the inflammatory response; therefore, they play an important role in the resistance of poultry to disease [20]. Heterophiles move toward foreign bodies and immediately phagocytize; however, they are unable to survive long in blood circulation; they last for 4–10 h, whereas they last for 1–2 days in tissues [21].

In this study, heterophiles were observed on the 28^th^ day after exposure, indicating a decrease in the number of heterophiles in C+ compared to C–. This is in accordance with a previous study by Khan *et al*. [10], which showed a decrease in heterophiles on day 21 following exposure to ochratoxin at a dose of 0.1 mg/kg. A previous study by Ricci *et al*. [12] also found a decrease in the number of heterophils and the occurrence of heteropenia following exposure to ochratoxin on day 14 at a dose of 1.4 mg/kg of ochratoxin feed. According to Khatoon and Ul Abidin [22], decreased leukocyte differentiation occurs due to the immunosuppressive action of ochratoxin and its inflammatory action on tissues, resulting in increased migration of leukocytes such as heterophils, eosinophils, and basophils into the tissues. Low levels of heterophils are found in the blood circulation.

There was a significant difference (p < 0.05) in the (C+) and (T2) treatments. At a dose of 1.6 g/kg (T2), the results were close to those of the negative control (C–), indicating that the administration of the mycotoxin detoxifier improved the impact of the mycotoxins. This shows that the effect of *Trichosporon mycotoxinivorans* and bentonite as an ingredient in the mycotoxin detoxifier in treatment (T2) at a dose of 1.6 g/kg of feed was able to reduce the adverse effects of mycotoxins so that the heterophile value was significantly different from the positive control (C+), which was not given mycotoxin detoxifier. *T. mycotoxinivorans* is a yeast cell isolated from the hindgut of the termite *Mastotermes darwiniensis* and has been investigated as having the potential to degrade mycotoxins, including ochratoxin [9]. In addition, bentonite is an adsorbent that attaches to aflatoxins in the digestive tract so that the aflatoxins are not absorbed by digestion [11]. This is in accordance with a previous study by Hanif and Muhammad [23], which showed that *T. mycotoxinivorans* contained in detoxifying agents can reduce the adverse effects of ochratoxin. Bentonite is an adsorbent capable of attaching to aflatoxins in the digestive tract so that aflatoxins are not absorbed by digestion [11]. Bentonite, which is included in the toxin binder, can effectively bind mycotoxin. Mycotoxin binders effectively bound mycotoxins in the feed [24]. This finding is supported by the research conducted [25], which showed that the materials of the mycotoxin binder tested could influence the effects of mycotoxin contamination in feed.

In the results of statistical analysis of the number of lymphocytes, the positive control group (C+) was significantly different from the negative control group (C–) for 28 days. Lymphocytes are part of the adaptive immune system [26]. Lymphocytes play an important role in the adaptive immune system [27]. Adaptive immunity arises after exposure to antigens from a pathogen [28]. Complete wound healing requires a complex reaction among immune cells, fibroblasts, epithelial cells, and endothelial cells [29]. Aflatoxin inhibited phagocytic and microbiocidal activity in chickens [30]. When one type of immune response is dysregulated, adaptive effects, such as delayed wound healing, tend to occur [29].

Lymphocytes are part of the white blood cells in the agranulocyte group. Lymphocytes play a role in response to antigens and stress by increasing antibody circulation [31]. When antigens enter the body, lymphocytes quickly respond to the immune system by stimulating and eliciting an initial response called the primary immune response [32]. In the results of this study, the number of lymphocytes increased in the positive control (C+), possibly due to delayed healing; therefore, the number of lymphocytes was still high on the day of blood sampling. The positive control group (C+) showed an increase in the number of lymphocytes when compared to the negative controls and the treatment group. Valtchev *et al*. [33] stated that the increase or decrease in the immune response in broiler chickens depends on the dose given and the duration of exposure to the toxin. Exposure to low doses of aflatoxin for a short period of time stimulates the immune system, whereas exposure to high doses for a longer period of time has an immunosuppressive effect [33]. In a previous study by Chen *et al*. [34], there was an increase in the number of lymphocyte cells in chickens exposed to aflatoxin for 21 days.

There was a significant difference (p < 0.05) in the (C+) and (T2) treatments. Treatment with a dose of 1.6 g/kg (T2) resulted in a decrease in the number of lymphocytes, which was close to that observed in the negative control (C−), indicating an improvement in the impact of mycotoxins by administering the mycotoxin detoxifier.

A significant difference (p < 0.05) was found between the positive control group (C+) and the negative control group (C–) of lymphocyte-heterophile ratio. This significant difference was possible because in the positive control group (C+), the number of heterophiles decreased and the number of lymphocytes increased, leading to a decrease in the ratio of heterophiles to lymphocytes. This is in accordance with the opinion of Thiam *et al*. [35] that a low ratio of heterophilic lymphocytes can be caused by a decrease in the level of heterophilic lymphocytes in blood circulation. In a study conducted in broiler chickens exposed to ochratoxin at a dose of 0.1 mg/kg for 21 days [10], there was a decrease in the number of heterophils and lymphocytes. Sugiharto *et al*. [36] explained that stress can cause heteropenia and lymphocytosis, resulting in a low ratio of heterophilic lymphocytes. This theory is in line with the results of this study, which showed a decrease in heterophiles and an increase in lymphocytes in chickens fed a stressor in the form of mycotoxin contamination, which resulted in a low heterophile lymphocyte ratio.

A significant difference (p < 0.05) was observed between the positive control (C+) and treatment with a *mycotoxin* detoxifier dose of 1.6 g/kg (T2). This significant difference is possible because the ingredients contained in the mycotoxin detoxifier dose of 1.6 g/kg can overcome the impact of mycotoxin contamination in the feed, which can be seen from the increase in the ratio of heterophilic lymphocytes (T2) approaching the negative control (C–). An increase in the lymphocyte-heterophil ratio indicates that the number of heterophils in the blood is high and the number of lymphocytes is low. The lymphocyte-heterophil ratio is used to measure the balance between non-specific immunity and the fast-acting and slower-acting responses of heterophils [37].

Mycotoxin detoxifier used in this study has a high affinity for effectively absorbing and modifying polar and non-polar toxins. In accordance with the findings of Khan *et al*. [10] that the binding of aflatoxin to the mycotoxin binder is based on the principle of polarity, whereby the negative polarity of aflatoxin is bound by the positive polarity of the toxin binder so that the toxin can be mobilized and eliminated from the animal’s body. Ochratoxin is a type of nonpolar molecule [8]. Mycotoxin modifiers, which can change the molecular structure of mycotoxins into less toxic or non-toxic metabolites [9], can be used as countermeasures against ochratoxins.

On other parameters, such as monocyte, MCH, and MCHC, treatment 2 at a dose of 1.6 g/kg was the best treatment, although there was no significant effect on the results. Monocytes are the phagocytic mononuclear cells that destroy and recycle tissue debris. The infiltration of monocytes during aflatoxicosis induces external trap formation of macrophages [38]. The phagocytic activity of heterophils and monocytes influences the antigen-presenting cells that mediate antibody synthesis [39]. In this study, monocytes did not exhibit a significant effect. Monocytes were not significantly different between the negative and positive controls because the results of SPSS analysis showed the same notation, indicating that there was no significant difference between treatment dose 2 and positive or negative controls, whereas there was a significant difference between treatment 1 and the negative control. MCH was used to determine the RBC’s Hb content; however, this result did not indicate a significant effect. In addition, no significant results were obtained for other parameters related to MCH, namely, MCHC. The MCHC was used to determine the RBC Hb concentration [40]; however, there was no statistically significant effect on this result. This insignificant result has also been reported by Umar *et al*. [3].

The number of erythrocytes decreased in the positive control. A decrease in the number of erythrocytes may be due to metabolic disorders caused by the presence of mycotoxins. The kidney is the organ most sensitive to synergistic effects of ochratoxin and aflatoxin [41]. Chronic kidney disease can disrupt the production of erythropoietin hormone, which plays an important role in erythropoiesis. Disruption of DNA synthesis due to mycotoxin metabolism (Vitamin B12 and folic acid deficiency) can also interfere with the formation of erythrocytes in the bone marrow [42]. Deficiency of nutrient absorption function in the digestive tract due to mycotoxins could be one of the causal factors causing a decrease in the average number of erythrocytes in mycotoxicosis sufferers, which is in line with the study of Ren *et al*. [43] that high levels of mycotoxin contamination can cause damage to the intestinal mucosa.

Hemoglobin levels decreased in the treatment group that received food contamination with mycotoxin. These results are consistent with the results of Kilany *et al*. [44], which showed that administration of aflatoxin in the 2^nd^ week produced an image of erythrocyte mass indicating anemia. Anemia can occur as a result of nephropathy, disrupting the process of erythropoiesis as well as disrupting protein metabolism as a result of hepatopathies. Decreased hemoglobin levels in this study could also be caused due to impaired Fe absorption due to decreased channel performance during mycotoxicosis [45].

On the basis of these data, the average number of leukocytes in the positive control group significantly differed from that in the negative control group, group treatment 1, and treatment group 2. The other three treatment groups, namely, negative control treatment, group treatment 1, and treatment 2, did not significantly differ from each other.

The number of leukocytes in the positive control group decreased. A decrease in the number of leukocytes can occur as a result of chronic infections caused by persistent mycotoxins entering the body, leading to increased inward migration of the leukocyte network, which can result in a decrease in the number of leukocytes inside blood vessels [46]. Changes in the number of leukocytes can also be attributed to the direct effects of ochratoxin in the germinal center of the lymphoid tissue and changes in immune response, which is in line with previous research showing that ochratoxin poisoning causes a decrease in lymphoid organ size [47].

The erythrocyte count, hemoglobin level, and the number of leukocytes increased in treatments 1 and 2 after administration of feed containing multi action toxin binder. This increase may be due to a combination mechanism of the ingredients contained in a multi action toxin binder, which works by adsorption and biotransformation. The adsorbent contained in the product is bentonite.

Dispersed in an aqueous medium has a negative charge on its surface as a result of isomorphic ion substitution metals with lower valence, so it can absorb a variety of biomolecules, such as mycotoxins, through cation exchange reactions [48]. This mechanism allows bentonite to act as a chemical sponge during channel digestion and secrete complexes of bentonite-aflatoxin bonds with feces [49]. The microorganisms contained in the product of this research as a biotransforming agent that degrades OTA in the gastrointestinal tract are yeast strains (yeast). This type of yeast is considered to be capable of degrading mycotoxins from toxic compounds to non-toxic compounds. OTA undergoes hydrolysis until it turns into Ochratoxin-α (OTα) by the carboxypeptidase produced by yeast [50]. OTα is a non-toxic OTA metabolite that is produced by intestinal flora and does not contain a phenylalanine group [51].

## Conclusion

Based on the results of the research and discussion, it can be concluded that the administration of mycotoxin detoxifiers at a dose of 1.6 g/kg (T2) had a significant effect (p = 0.05) on increasing the number of heterophils and the ratio of heterophil lymphocytes, leukocytes, erythrocytes, and hemoglobin of broiler chickens fed mycotoxin-contaminated feed (aflatoxin-B1 and OTA) for 28 days.

## Authors’ Contributions

MWJ, FH and ES: planned and designed the study. HP, SD, and GJ: Revised the research plan and designed the study. MWJ and FH: Conducted the research and drafted the manuscript. ES, ML, and TDL: Supervised the study. BS, MA, SS, RS, and SU: Analyzed the data and reviewed critically the manuscript for important intellectual content. All authors have read, reviewed, and approved the final manuscript.
